# Walking the tight rope of DEI implementation: paradox mindset and emotional capabilities as preconditions for middle managers' success

**DOI:** 10.3389/fpsyg.2024.1396512

**Published:** 2024-06-03

**Authors:** Smaranda Boroş, Andreea Gorbatai

**Affiliations:** ^1^Vlerick Business School, Ghent, Belgium; ^2^Ghent University, Ghent, Belgium; ^3^University of Stellenbosch Business School, Stellenbosch, South Africa

**Keywords:** paradox mindset, middle management, DEI implementation, leadership skills, organizational paradoxes, emotional capability

## Introduction

In the competitive landscape of modern organizations, middle managers are inherently subjected to immense pressure. Changes in organizational goals and priorities not only threaten but also transform their roles and identities (Thomas and Linstead, 2002), imposing high emotional work demands during the implementation process (Clarke et al., 2007). Occupying a unique structural position, middle managers find themselves at the forefront of strategic change. They are both targets, as their “strategic importance in the social system” of the organization is altered (Van Doorn et al., 2023), and agents of change, tasked with implementing organizational strategy in day-to-day operations (Harding et al., 2014).

As organizations transition to new structures, processes, and technologies, middle managers are the recipients of top-down expectations to facilitate team adaptability and sense-making (Luscher and Lewis, 2008), and implement organizational strategy, while being expected to report on progress toward the expected goals, and champion potential alternatives (Floyd and Wooldridge, 1992; Mantere, 2008). Among all these challenges of being the “boots on the ground” of organizational change, one category of change implementation stands out: DEI strategy execution. The implementation of diversity, equity, and inclusion strategy is a particularly treacherous type of organizational change for middle managers, as it introduces two additional sources of resistance: identity-related intrapersonal and interpersonal tensions (Gorbatai et al., 2021), and paradoxes arising from balancing multiple metrics of organizational performance.

In this opinion paper, we draw on the Leading Diversity (LeaD) model (Homan et al., 2020) to highlight two leadership skills that middle managers, as a uniquely positioned type of leaders working with diversity, can employ when dealing with DEI-related change and its accompanying tensions. LeaD is a functional diversity leadership model that identifies the skills - such as cognitive understanding, social perceptiveness and behavioral flexibility (the latter being mediated by the former two) - needed for success in managing diversity-related work. In applying the leadership skills the model proposes to the middle management position, we discuss how both a cognitive understanding perspective based on a paradox mindset and a social perceptiveness approach rooted in interpersonal and intrapersonal emotional capabilities are important skills a middle-manager can draw upon for a smooth implementation of DEI strategy.

We leverage various academic sources to outline the challenges that middle managers encounter when putting DEI policies into action, and the core capabilities they need to succeed. On the one hand, drawing from organizational change studies, we pinpoint multiple balancing acts expected from middle managers during change implementation efforts. On the other hand, building on diversity and leadership research, we identify the context-specific capabilities crucial for effective diversity management. We further delve into these competencies, tailored to middle managers' unique hurdles: utilizing paradox theory to clarify the advantages of a paradoxical mindset and how to cultivate it, and referencing organizational emotions studies to refine the necessary emotional skills and their cultivation. By synthesizing insights across these fields and aligning them with middle managers' challenges, we provide evidence-based, targeted guidance on critical skills required to effectively navigate DEI initiatives and strategies for their development.

## A paradox mindset

DEI-related paradoxes occur at three levels: personal (the ones managers themselves experience - like autonomy), group level, and organization-related (Luscher and Lewis, 2008). On a personal level, managing ongoing, team-level performance goals and objectives while monitoring and improving the team's implementation of DEI initiatives poses pressures and paradoxical challenges on middle management (Gorbatai et al., 2022). As part of DEI strategy implementation, certain processes might be automated, or at least standardized to remove managerial subjectivity and reduce the risk of bias. In this context, similar to other automation changes, managers can find their prior autonomy is restricted (Raisch and Krakowski, 2021), such that decisions like hiring or task allocation, where they could previously rely on their experience and knowledge of team-members are now removed from their purview, reducing their control and authority over their teams. In addition to the tension between autonomy and control, the automation or standardization of people-related tasks in the attempt to reduce the human decision-making variability and bias is bound to elicit more resistance compared to the automation of other tasks, such as financial reporting, due to middle managers' beliefs about their own people-related skills a team leaders, and to fears of peer's judgment if their decision-making and leadership is otherwise constrained (Nolan and Highhouse, 2014; Neumann et al., 2023).

At the team level, middle managers' cognitive understanding of diversity is important for knowing the “favorable and unfavorable processes that can be instigated by diversity” (Van Knippenberg et al., 2004). It is important to recognize that favorable (i.e., information elaboration) and unfavorable (i.e., intergroup bias) processes can unfold simultaneously: it is not always a clear-cut task to foster the cognitive elaboration processes and move away from the intergroup bias ones. Instead, in a team where paradoxes are managed, people can be simultaneously aware of the benefits and drawbacks of diversity; intergroup dynamics can simultaneously acknowledge the need to rebalance the inequities in a system and the sense of continued injustice from marginalized groups with the fear of loss of power and opportunities from those having privilege.

In line with research on most effective framings for DEI implementation success (Thomas and Ely, 1996), team leaders are essential for managing group-level paradoxes to enable a learning-and-effectiveness paradigm based on the integration of diverse members. Specifically, managers can lead change by openly addressing team members' fears and misconceptions as they relate to the DEI initiatives and thus allowing their team to safely learn and explore the avenues for change; while also decisively continuing to address systemic inequalities in the organization and, through their actions, effectively advancing the DEI strategy. This approach fosters a climate of cultural inclusion in the team (Chavez and Weisinger, 2008), such that fears, resistance, and doubts on all sides are actively managed as the team progresses on its diversity goals.

Lastly, on the organizational level, managers are subject to tensions between investment in actions connected to hard-to-achieve short-term financial results required for the organization to perform, as compared against its competitors, and investment in DEI-related behaviors expected to generate long-term benefits for the team and organization, such as spending additional time and resources recruiting a new team member from a minority background or taking into consideration all voices on their team prior to making a decision. Even when managers are aware of the importance of equity and inclusion for capitalizing on the unique perspectives of a diverse workforce (Chavez and Weisinger, 2008; Boroş and Gorbatai, 2023) and personally value such behaviors, managers experience a paradox in the attempt to balance the short-term, result-focused actions with long-term investment in a more diverse and inclusive team climate.

This is why middle managers working on DEI issues must embrace a paradox mindset, in order to successfully work through these tensions. A paradox mindset refers to “the extent to which one is accepting of and energized by tensions” (Miron-Spektor et al., 2018), such that instead of favoring one demand or process over the other, one views tensions as a chance for growth and learning. Embracing the paradox mindset means acknowledging and adapting to the ongoing tensions of conflicting demands, rather than trying to eliminate them. It's about shifting from having to pick one option over another to learning how to continuously manage their demands (Rubin et al., 2023). This mindset encourages people to switch between exploration and exploitation, which, in turn, motivates employees to engage in more innovative behaviors (Liu and Zhang, 2022). Miron-Spektor and collaborators offer three strategies to cultivate a paradox mindset: (1) reframe the question (i.e., from a choice to a “how could both options be pursued”); (2) accept the tension and develop comfort with the discomfort, and (3) distance yourself and search for new possibilities. We see then that a prerequisite of working with a paradox mindset is to have good emotion regulation skills. We will elaborate on this next dimension next.

## Emotional capabilities

One visible side of emotional dynamics linked to DEI initiatives concerns the plethora of emotional dynamics that diversity brings along in groups and organizations. Such emotional dynamics can escalate into conflicts and prevent the richness of diversity from materializing. But “when leaders are able to make a correct prognosis regarding the diversity-related process that is most likely to become dominant in a team they can anticipate which behavior is most likely to be effective in proactively shaping the diverse team's processes in a way that intergroup bias is avoided, or information elaboration is invited” (Homan et al., 2020, p. 1114). Leaders can better read these situations and act accordingly if they have well-developed emotional capabilities (Schlegel and Mortillaro, 2019; Homan et al., 2020) - i.e., emotional awareness (the ability to recognize and understand these emotions – Joseph and Newman, 2010) and regulation (the ability to “influence which emotions they have, when they have them, and how they experience and express these emotions” - Gross, 1998, p. 275).

The burden of emotion work does not need however to fall square on the leaders' shoulders alone. A different avenue to support smoother team dynamics is to build emotion work capacity in teams – i.e., develop collective emotional intelligence. A team's capacity to be aware of (Boroş and Vîrgǎ, 2020) and work with (Curşeu et al., 2012) emotions can prevent conflict escalation (Boroş, 2020), or can lead to better conflict management strategies (Boroş et al., 2017) when conflict erupts. In both cases, this capacity further impacts group effectiveness and creativity (Curşeu et al., 2015). Research shows that the simple practice of emotion awareness in teams (Boroş and Curşeu, 2013) and organizations (Brouwer and Boroş, 2010) appears to be as powerful an intervention as cultivating a mindset of openness to diversity. Working with the emotions that are right in front of us is step one toward building resilience and inclusion in diverse organizations.

How can managers influence the development of collective emotional intelligence? Research has long proven now how fostering certain group norms leads to developing teams' emotional capabilities (Druskat and Wolff, 2001). Examples of such norms are practicing perspective taking and reciprocal understanding – to develop emotional awareness capabilities, or, confronting norm breakers and showing caring for group members – to develop emotional regulation capabilities (Druskat and Wolff, 2001). Managers can actively and consistently make room to hear minority voices (awareness), and advance and reinforce (by calling out norm breakers and praising good practices) norms leading to inclusion of all members (regulation), and overall, by nurturing a climate of psychological safety (Edmondson and Lei, 2014) that support healthy task-related divergence of opinions and debates without allowing them to escalate to interpersonal conflicts.

While the Leading Diversity (LeaD) model emphasizes the emotional capabilities required for leading a team and managing interpersonal dynamics, middle managers also contend with the internal emotions stirred by the many paradoxes they manage and reconcile within their teams and organizations. Such emotions unfold on two dimensions: one related to the proactive stance of pushing for change (Homan et al., 2020), and the other, as a reactive response linked to the recognition and acceptance of their contribution to the faulty status-quo. From a proactive perspective, middle managers must grapple with complex emotions such as frustration, anxiety, and even anger arising from the tension between accomplishing short-term and long-term goals, balancing people-performance objectives and autonomy vs. control, and the dichotomy of engaging in tasks where one is an expert (i.e., the functional position of a middle manager) vs. grappling with projects where one is a novice (i.e., diversity initiatives).

From a reactive perspective, the other paradox inherent to the internal emotional dynamics of managers working on DEI is the desire to do good combined with the awareness of one's own privilege. Research shows that when individuals confront their own privilege and learn about inequalities, they often experience a range of emotions such as shame, guilt, and fear of losing power. If these emotions are suppressed instead of being recognized and confronted, resistance emerges as a coping mechanism (Thomas and Plaut, 2008). Actively inhibiting the observable expression of the emotional experience (Gross and Thompson, 2007) shields us from the short-term pain of confronting unpleasant realities. However, it can also isolate managers (Boroş et al., 2019), rendering them unable to form the connections needed (Boroş and Van Gorp, 2017) to support their teams in working through the emotional issues that diversity brings.

In the context of acknowledging one's privilege, emotional capabilities such as awareness (Joseph and Newman, 2010) and regulation (Gross, 1998, p. 275) allow for the possibility to choose more effective responses to deal with the complex emotional dynamics elicited by diversity. Specifically, one particular technique of emotional awareness has been shown to be effective in these situations: affirmative introspection, “the ability to take an honest look inward, with curiosity in a non-judgmental way. It involves the ability to gain insights into the multiple layers of your experiences and to accept what you see, both your strengths and your vulnerabilities” (Gardenswartz et al., 2010).

In summary, the emotion work that middle managers are invited to do in order to support the diversity-related processes in teams can be done by advancing norms that foster their own teams' collective emotional capabilities (i.e., awareness and regulation) and by relying on, and developing their own emotional capabilities.

## Conclusions

This opinion paper proposes solutions to the unique challenges that middle managers face in implementing DEI strategies in organizations, including the pressure to balance multiple organizational goals, the need to facilitate team adaptability, and the responsibility to implement and report on strategic initiatives. This opinion paper emphasizes the importance of two key leadership skills for managing DEI-related change: a paradox mindset and emotional capabilities. A paradox mindset allows managers to reconcile the tensions and paradoxes inherent in DEI implementation, such as balancing short-term profit metrics with long-term DEI aspirations. Emotional capabilities, such as awareness and regulation, enable managers to effectively navigate the complex emotional dynamics elicited by diversity. These can be developed at both individual level (e.g., through affirmative introspection) or within the team (e.g., by fostering norms that develop collective emotional intelligence). This work applies two core skills of the LeaD diversity leadership model to the context of middle managers and expands on these skills with insights from related research. This is an important contribution to diversity-related change in organizations, as it focuses on how middle managers can best navigate their emotional and cognitive challenges of their organizational roles (Thomas and Linstead, 2002; Clarke et al., 2007), by working with a paradox mindset and fostering change-related emotional capabilities within themselves and their teams. The insights offered in this paper provide valuable guidance for middle managers seeking to effectively implement DEI strategies in their organizations and for LD consultants who design DEI trainings targeted at middle managers.

## Author contributions

SB: Writing – review & editing, Writing – original draft, Conceptualization. AG: Writing – review & editing, Writing – original draft, Conceptualization.
